# Exercise‐induced effects on the metabolome of endurance and strength‐trained athletes in comparison with sedentary subjects: A pilot study

**DOI:** 10.14814/phy2.70206

**Published:** 2025-02-04

**Authors:** Mario Parstorfer, Gernot Poschet, Kirsten Brüning, Birgit Friedmann‐Bette

**Affiliations:** ^1^ Department of Sports Medicine, Medical Clinic VII University Hospital Heidelberg Heidelberg Germany; ^2^ Olympic Training Center Heidelberg Germany; ^3^ Center for Organismal Studies, Heidelberg University Heidelberg Germany

**Keywords:** athletes, endurance training, exercise metabolome, phenotype, strength training

## Abstract

Little is known about the exercise‐induced adaptations of the metabolome in endurance and strength athletes in comparison with sedentary subjects. In order to analyze exercise‐induced effects, quantitative, targeted metabolomics (Biocrates MxP® Quant 500) were performed in plasma samples before and after one bout of endurance or resistance exercise (RE) in 12 strength‐trained weightlifters (ST), 10 endurance‐trained runners (ET) and 12 sedentary controls (CG) at the end of each of three characteristic training phases. Performance and anthropometric data were significantly different between CG and athletes. A significant exercise‐induced increase in lactate (Lac) was observed in all groups after all exercise tests. After endurance exercise (EE), there were significant increases in acetylcarnitine, arachidonic acid, and docosahexaenoic acid in CG and ET while aconitic acid, hippuric acid, glutamate, hexoses, xanthine were significantly increased in ET only. Only CG showed increases in several triglycerides following EE. RE, however, induced significant increases in Lac only. In summary, EE induces distinct increases in some metabolites of the fatty acid metabolism and the oxidative defense system in ET and CG. There are some indications for specific adaptations of the energy metabolism after long lasting endurance training with a distinct exercise‐induced response of the metabolome in ET.

## 1 INTRODUCTION

Targeted metabolomics has emerged as a tool for the precise measurement of metabolic adaptations in health and disease. Some recent investigations revealed characteristic differences between the resting metabolome of athletes in various sports and compared to untrained subjects (Al‐Khelaifi et al., 2018; Parstorfer et al., 2023; Sakaguchi et al., 2019; Schranner et al., 2021). Furthermore, there is special interest in the metabolic response pattern to acute bouts of aerobic and anaerobic exercise. Exercise‐induced changes of the plasma metabolome were investigated after endurance exercise (EE) lasting between 10 min (cycle ergometer test) and 10:40 h (simulated treadmill ultramarathon) applying targeted (Nemkov et al., 2023; Pellegrino et al., 2022; San‐Millán et al., 2020; Schader et al., 2020) or untargeted metabolomics (Howe et al., 2018; Lehmann et al., 2010; Manaf et al., 2018; Morville et al., 2020; Stander et al., 2018). Studies on the effects of resistance exercise (RE) on the plasma metabolome are scarce (Morville et al., 2020; Pellegrino et al., 2022). Recently, the question arose if in high performance sports exercise‐induced alterations of the blood metabolome might allow the identification of plasma metabolites which can be used for monitoring athletic performance during training and competition (Nemkov et al., 2023; San‐Millán et al., 2020). We wondered if characteristic molecular signatures might emerge when endurance‐trained athletes, strength‐trained athletes and sedentary subjects are subjected to specific standardized exercise bouts.

At present, the current athletes' physical status is determined by various exercise tests (Al‐Khelaifi et al., 2018; Bosquet et al., 2002; McMaster et al., 2014). Spiroergometry and the determination of lactate (Lac) thresholds are the most common methods for the assessment of the aerobic performance capacity. For the evaluation of skeletal muscle strength, diverse tests are applied: in dynamic settings, one‐repetition maximum (1RM) tests and isokinetic dynamometer tests; in static settings, maximal isometric strength tests (MVIC) (Lac & Maso, 2004; McMaster et al., 2014; Yan et al., 2009). However, none of these tests sensitively detects physiological differences in the resting metabolism of athletes and non‐athletes or in response to an exercise stimulus (Al‐Khelaifi et al., 2018; Bogdanis, 2012; Yan et al., 2009). To the best of our knowledge, there are only four studies which investigated in metabolic patterns applying metabolomics in differently trained athletes: Differences in the basal plasma metabolome and its response to a bicycle ramp test between endurance athletes, sprinters, natural bodybuilders and in untrained subjects were reported (Schranner et al., 2021). One study observed diverse metabolic profiles in saliva samples of basketball players with different player positions (Khoramipour et al., 2021). Distinct metabolic profiles in serum samples obtained during doping tests in high‐power and high endurance athletes were described (Al‐Khelaifi et al., 2018). Our research group detected differences in the resting plasma metabolome between endurance‐ and strength‐trained athletes as well as between sedentary subjects and both groups of athletes (Parstorfer et al., 2023).

The aim of the present study was to find out if there are characteristic changes of the plasma metabolome in response to a standardized bout of EE (in endurance‐trained athletes and in sedentary subjects) or to a bout of RE (in strength‐trained athletes and in sedentary subjects). Furthermore, as training varies a lot throughout a year with periods of high volume—low intensity training (preparation phase) in contrast to high intensity—low volume training (competition phase [CP]) or very low volume—low intensity training (regeneration phase [RP]) we wondered if the different metabolic challenge during these training phases might be reflected in the plasma metabolome.

## 2 MATERIALS AND METHODS

### 2.1 General design

This study is part of an investigation on the differences in the metabolome between endurance‐trained athletes, strength‐trained athletes and sedentary subjects. Detailed subject data, the data of the basal metabolome and changes in the basal metabolome of strength‐trained athletes, endurance‐trained athletes and sedentary subjects throughout 1 year are provided elsewhere (Parstorfer et al., 2023). All subjects provided written and informed consent prior to participation in the study. The project was given approval by the Ethics Committee of the Medical Faculty of the University of Heidelberg (S513/2018), and it was carried out in compliance with the Helsinki Declaration. The study was preregistered (DRKS00015511) at the German Clinical Trials Register.

In brief, 12 male strength‐trained athletes (ST), 10 male endurance‐trained athletes (ET) and 12 male sedentary subjects (control group, [CG]) first performed preliminary testing for determination of the anthropometric data and for measurement of the maximal performance and endurance capacity after passing a health check. All the participants were Caucasians. They performed an incremental exercise test to exhaustion either on a treadmill (ET and CG, ELG70, Woodway USA Inc., Waukesha, USA) or on a cycle ergometer (ST, Excalibur Sport, Lode BV Medical Technology, Groningen, Netherlands) to assess cardiorespiratory performance. Treadmill spiroergometry started with a 1‐minute warm‐up at 4 km · h^−1^. Then, running velocity (start at 6 km · h^−1^, incline 1.5%) was increased by 2 km · h^−1^ every 3 min until volitional exhaustion, followed by 5 min of recovery at 4 km · h^−1^ and 5 min of passive rest. Cycling spiroergometry started at 50 W. Load was increased by 50 W every 3 min until volitional exhaustion, followed by 5 min of recovery with 50 W. Exhaustion was considered if at least two of the following criteria were met: high levels of blood Lac concentration (BLa; >8‐ mmol · L^−1^); a plateau in oxygen uptake (VO_2_) despite increasing work rate; a respiratory exchange ratio (RER) above 1.1.

During each test, oxygen uptake (VO_2_), carbon dioxide release (VCO_2_), and ventilation (VE) were recorded with a breath‐by‐breath spirometry system (Geratherm Respiratory GmbH, Bad Kissingen, Germany) and the corresponding software Blue Cherry (version 1.3.0.5, Geratherm Respiratory GmbH, Bad Kissingen, Germany) using an individual adjusted face mask. Before each test, both sensors were calibrated with known gas concentrations and the flowmeter with a 3 L‐syringe according to the manufacturer's instructions. Heart rate (HR) was recorded continuously with a 12‐lead ECG using the Amedtec ECGpro Software (version 4.21.0, AMEDTEC Medizintechnik Aue GmbH, Aue, Germany) and self‐adhesive electrodes in all three groups. Maximum oxygen uptake (VO_2_max) and RERmax were detected as the highest 30 s average values at the time of volitional exhaustion. If participants did not finish the entire increment, their maximal running velocity or cycling performance was linearly interpolated.

In ET and CG, capillary blood samples (20 μL) from the earlobe were taken within the 30 seconds break period for Lac measurement (Biosen S‐line, EKF Diagnostic, Barleben Germany). Blood Lac values at various running velocities were processed with the Winlactat® software (version 4.0, mesics GmbH, Munster, Germany). The 4 mmol · L^−1^ Lac threshold (LT_4_) and an individual anaerobic threshold (IANT) were determined. IANT was defined as the running velocity associated 1,5 mmol · L^−1^ above the mean of the three lowest Lac values (Heck & Beneke, 2008).

### 2.2 Experimental design

**FIGURE 1:**
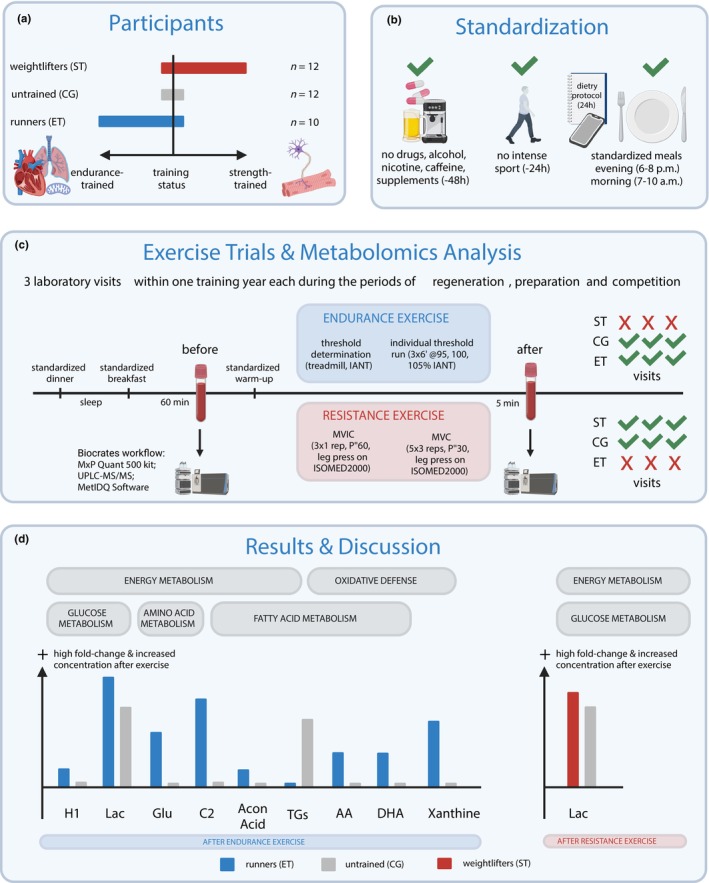
Schematic overview of the study. (a) Strength‐trained (weightlifters, ST, 12 males) and endurance‐trained athletes (runners, ET, 10 males) as well as healthy sedentary subjects control group, CG, 12 males) were recruited. Bars reflect the dominant training status within each group. (b) Dietary control 24 h before exercise trials. Subjects had to refrain from drugs, alcohol, nicotine, caffeine and supplements 48 h before each test. They had to avoid intense physical activity or training 24 h before each test. All participants consumed a standardized dinner the day before each test and a standardized breakfast on each test day. (c) All participants performed three laboratory visits within one training year each during the periods of regeneration, preparation and competition. The EE was only performed by ET and CG, whereas RE was performed by ST and CG. EE consisted of an individual anaerobic threshold (IANT) determination followed by a run of 3 × 6 min at 95%, 100% and 105% of predetermined IANT. RE consisted of 3 × 1 repetition of a maximum voluntary isometric contraction (MVIC) followed by 5 × 3 repetitions of a maximum voluntary contraction (MVC), both on the same leg press. 60 min before and 5 min after each exercise trial venous blood samples were taken from the forearm vein. Sample treatment and analysis by UPLC–MS/MS and MetIDQ Software according to Biocrates MxP Quant 500 kit workflow. Data treatment and statistical analysis were performed in the software R and MetaboAnalyst. (d) Regular endurance or resistance training induces systematic changes in the concentration of several metabolites in athletes in response to a specific exercise and throughout 1 year of training associated with adaptations in overall energy metabolism as well as oxidative defense system. Figure created with BioRender.com.

All measurements in ET and ST were performed at the end of each of three characteristic training phases: a preparatory phase (PP) with high‐volume and low‐intensity specific training sessions building foundational fitness, a CP with regular competitions as well as low‐volume, high‐intensity specific workouts to maximize performance in competitions, and a transition or RP with either no training at all or low‐volume, low‐intensity unspecified workouts allowing for recovery (Bompa & Buzzichelli, 2015). Before the measurements, participants had already trained in PP and CP for at least 6 weeks. Laboratory tests in CP were spaced out from the main competition by 3 ± 2 weeks. In each training phase, ET underwent one EE test, ST one RE test, while CG completed both exercise tests three times throughout the study phase. Control subjects completed three testing sessions aligned with the athletes' training phases (preparatory, competition, and regeneration). A minimum of 96 h and a maximum of 14 days separated each session, with consistent intervals in all tests for each subject. Control subjects were not physically matched to the athletes but followed the same testing schedule to assess exercise‐induced metabolic responses in non‐athletic individuals and completed the exercise tests in a randomized order. Figure 1 provides a schematic representation of the experimental design. Figure 1 Here or use as abstract figure.

### 2.3 EE

The EE consisted of (1) a submaximal stepwise increasing treadmill test for determination of the actual IANT and (2) of 3 × 6 min threshold runs based on the determined thresholds for ET and CG (Figure 1). On each occasion, both protocols were performed separated by 60 min passive rest. First, running threshold determination was performed as the same incremental exercise test on treadmill described above, but only with a maximum intensity slightly above 4 mmol · L^−1^ Lac threshold (LT4). Treadmill tests and threshold runs are regularly applied in high‐performance endurance sport in Germany to assess running velocity at the maximal Lac steady state. Test duration was 19.7 ± 1.5 min in ET and 11.3 ± 1.4 min in CG. Part two consisted of 3 × 6 min running at velocities of 95%, 100% and 105% v_IANT_ (v_95_, v_IANT_, v_105_) interspaced by 30 seconds of rest. HR was continuously recorded (M400, Polar Electro Inc., Kempele, Finland) with the mean of the last 15 s in each stage being used for HR calculation of the corresponding stage. Capillary blood samples were taken immediately at the end of each stage rest as well as 1, 3 and 5 min after the individual threshold run. After 5 min of rest after the last run, the blood samples for metabolomic analysis were collected. Rate of perceived exertion values from 6 (very very light) to 20 (very very hard) were obtained immediately after the individual threshold run (Borg, 1970). The energy cost of the activity (metabolic equivalent, MET) was determined by the mean of the relative oxygen cost of preliminary testing (mL · min^−1^ · kg^−1^) at the three corresponding velocities (95%, 100% and 105% v_IANT_) of the threshold run each multiplied by 3.5.

### 2.4 RE

The RE consisted of a set of strength tests: a maximum isometric strength test and a maximum dynamic strength protocol in double leg press on a computer‐driven isokinetic device with corresponding leg press module (IsoMed 2000; D&R Ferstl, Hemau, Germany) for ST and CG (Figure 1). The maximum isometric strength test consisted of three maximal isometric voluntary contractions each over a period of 3 s (MVIC) in double leg press with 1 minute rest between the repetitions. Subjects were seated upright with 90° hip flexion, 120° knee flexion (0°, full knee extension) and their arms folded across the chest. The maximum of the three MVIC values was used for further analysis.

The second part of RE was performed as a maximal concentric dynamic strength test (MVC). Five sets of three repetitions at 75 mms^−1^ of the same exercise were performed by the participants with 30s rest between sets, 10–90° knee flexion (0°, full knee extension), seated upright with 90° hip flexion and their arms folded across the chest. For all tests, the participants were given a standardized verbal instruction with visual and auditory feedback to ensure maximal effort during each repetition and set. Both trials were separated by 5 min of passive rest.

### 2.5 Standardization

As already reported elsewhere, participants were asked to refrain from any drugs (e.g., nicotine, alcohol, medication, caffeine) and soft drinks or special teas (green or black tea) 48 h before each laboratory visit (Parstorfer et al., 2023). Nutritional supplementation (e.g., creatine, beetroot) was not allowed 48 h before each visit. All participants were asked to avoid intense physical activity and training 24 h before each visit (Figure 1). Participants were asked to not change their style of living (including mode of transportation) during the study. It was not possible to test our group of athletes in a fasted state due to the testing procedure and duration of the exercise tests and because of testing them in their competitive phase. Therefore, all participants were tested in non‐fasted state with standardized meals in the evening and morning before the exercise tests. Each meal consisted of an equal nutrient distribution (55%–60% carbohydrates, 25%–30% proteins, 13%–17% fat). To prevent possible bias, meal size and meal time were kept the same throughout the study (Parstorfer et al., 2023).

### 2.6 Sample handling

Venous blood samples (single 4.9 mL tube, EDTA, S‐monovette, Sarstedt, Sarstedt, Nümbrecht, Germany) were taken from the forearm vein of all participants in a seated position: (i) 60 min postprandial and before warm‐up of the consecutive exercise, (ii) 5 min after exercise. After collection the tube was immediately stored in crushed ice (4°C) for no longer than 2 h. Tubes were then centrifuged at 4000× *g* at 4°C for 10 min. After separation supernatant plasma was instantly aliquoted, snap frozen in liquid nitrogen and stored at −80°C until analysis.

### 2.7 Targeted metabolomics analysis

The Biocrates MxP® Quant 500 kit (Biocrates Life Sciences AG, Innsbruck, Austria) can be used for analysis of up to 630 metabolites from 26 compound classes of widely different structures and polarities. Compound classes include lipids like acylcarnitines (Cx:y), hydroxylacylcarnitines [C(OH)x:y] and dicarboxylacylcarnitines (Cx:y‐DC), lysophopatidylcholines, phosphatidylcholines, sphingomyelins (SMx:y) and sphingomyelin derivatives [SM(OH)x:y], ceramides and derivatives (cer‐, hexcer‐, hex2cer‐ and hex3cer‐), cholesteryl esters, and diglycerides and triglycerides (the first fatty acid is counted individually, in the case of three fatty acids, the last two fatty acids are summed), which are all measured by FIA‐MS/MS, as well as amino acids, amino acid‐related com‐pounds, bile acids, biogenic acids, biogenic amines, the sum of hexoses (H1), p‐cresol sulfate, carboxylic acids, fatty acids, hormones and related metabolites (abscisic acid, cortisol, cortisone, dehydroepiandrosterone sulfate; DHEAS), indoles and derivatives (indole, 3‐indoleacetic acid, 3‐indolepropionic acid, indoxyl sulfate), xanthine and hypoxanthine, choline, trigonelline, and trimethylamine N‐oxide (TMAO), which are determined by UPLC–MS/MS.

In brief, 10 μL of human plasma were pipetted on a 96 well‐plate containing internal standards and dried under a nitrogen stream using a positive pressure manifold (Waters). A total of 50 μL of a 5% phenyl isothiocyanate (PITC) solution was added to each well to derivatize amino acids and biogenic amines. After 1 h incubation at room temperature, the plate was dried again. To extract the metabolites, 300 μL 5 mM ammonium acetate in methanol was pipetted to each filter and incubated for 30 min. The extract was eluted into a new 96‐well plate using positive pressure. For further LC–MS/MS analyses, 150 μL of the extract was diluted with an equal volume of water. For FIA‐MS/MS analyses, 10 μL extract was diluted with 490 μL of FIA solvent (provided by Biocrates). After dilution, LC–MS/MS and FIA‐MS/MS measurements were performed. For chromatographical separation, an UPLC I‐class PLUS (Waters) system was used coupled to a SCIEX QTRAP 6500+ mass spectrometry system in electrospray ionization (ESI) mode. Data was generated using the Analyst (Sciex) software suite and transferred to the MetIDQ software (Biocrates Life Sciences AG), which was used for further data processing and analysis. All metabolites were identified using isotopically labeled internal standards and multiple reaction monitoring (MRM) using optimized MS conditions as provided by Biocrates. For quantification, either a seven‐point calibration curve or one‐point calibration was used depending on the metabolite class. Sample orders were randomized to ensure that the results obtained are not influenced by the order of analysis.

### 2.8 Statistical analysis

Data preprocessing and analysis steps were performed using R (version 4.0.3, R Core Team, Vienna, Austria) (R Core Team, 2020) and the web‐based tool MetaboAnalyst 5.0 (https://www.metaboanalyst.ca) (Pang et al., 2021). Data quality was ensured by a multiple step procedure, which is presented elsewhere (Parstorfer et al., 2023). The final data matrix (367 metabolites and 34 samples) was used for further analysis regarding group (ST, ET, CG), exercise (pre, post) and training phase (PP, CP, RP). The CG was matched to both groups of athletes (matched to ET, CGE; matched to ST, CGS) separately and visited the laboratory twice at each training phase. When necessary, both control conditions were merged into a single CG by calculating the mean metabolite values for each control subject individually, prior to the corresponding exercise test at the same time point. All data were tested for normality of distribution using Shapiro–Wilk procedure. Repeated measures analysis of variance (ANOVA) was used for comparison of subject characteristics with log‐transformed data: group (ST, ET, CG), exercise (pre, post) and training phase (PP, CP, RP) with controlling for the effects of breakfast. Significant main effects were followed by pairwise comparisons with Bonferroni correction. For all statistical tests level of significance was set at Bonferroni adjusted *p* < 0.05. Data are presented as mean ± standard deviation (SD). Volcano plots with log2 fold‐changes cut‐off values (log2FC ≤ −1 & log2FC ≥1) were used by default to provide an overview of decreased and increased metabolite concentrations. Multivariate analysis were used to maximize the variance in the metabolic profiles between groups, exercise and training phase by using MetaboAnalyst 5.0 (https://www.metaboanalyst.ca) (Pang et al., 2021). As single metabolites could be active in multiple pathways multivariate analysis (log‐transformed, auto‐scaled data) was used to correct for heteroscedasticity, to reduce skewness of the data and to reduce mask effects (van den Berg et al., 2006). Metabolites responsible for differences were identified using partial least squares discriminant analysis (PLS‐DA) and variable importance in the projection (VIP) (Gromski et al., 2015; Szymańska et al., 2012). Thus, potential different metabolites between the groups and time points and to rank the metabolites according to their importance could be identified. VIP >1.5 was considered sufficient for discrimination (Weljie et al., 2011). The quality of the PLS‐DA model was estimated with 10‐fold cross validation method by goodness of fit (R^2^) and ability of prediction (Q^2^cum). The significance of class discrimination was assessed by permutation tests with 100‐random permutation cycles due to the small sample size (Zhong et al., 2012). We used a combination of uni‐ and multivariate statistical analysis to strengthen the results. When appropriate, simultaneous comparison of uni‐ and multivariate results were presented with Venn diagrams. Features were regarded as robust if univariate analysis revealed them as significant (*p* ≤ 0.05) with concomitant VIP ≥1.5 in multivariate analysis.

## 3 RESULTS

### 3.1 Characteristics of study participants

ST were significantly (*p* < 0.05) younger compared with CG and ET while there were no differences in height. ET weighed less with concomitant less bodyfat compared to CG and ST (*p* < 0.05) (Table 1).

**TABLE 1:** Characteristics of study participants.

	Control (CG)		Endurance‐trained (ET)		Strength‐ trained (ST)	
	(*n* = 12)		(*n* = 10)		(*n* = 12)	
Age (years)*	24.8 ± 4.2^S^		24.0 ± 2.9^S^		20.2 ± 2.6^C^, ^E^	
Height (cm)	180.8 ± 8.8		176.8 ± 6.8		175.0 ± 8.7	
Weight (kg)*	81.9 ± 18.4^E^		66.5 ± 9.2^C^, ^S^		80.3 ± 13.0^E^	
Body fat (%)*	14.9 ± 6.5^E^		8.3 ± 1.4^C^, ^S^		10.6 ± 4.3^E^	
VO_2_max (L · min^−1^)*	3350 ± 515^E^		4316 ± 554^C^, ^S^		3583 ± 767^E^	
VO_2_max (mL · min^−1^ · kg^−1^)*	41.2 ± 6.5^E^		65.1 ± 4.8^C^, ^S^		42.5 ± 4.7^E^	
VE (L · min^−1^)*	118 ± 11^E^		156 ± 26^C^, ^S^		122 ± 23^E^	
HR (bpm)*	196 ± 10^S^		186 ± 10		183 ± 15^C^	
RER (VCO_2_ · VO_2_^−1^)	1.14 ± 0.05		1.15 ± 0.05		1.15 ± 0.06	

*Note*: All data are presented as mean ± SD.

Abbreviations: VE, maximum ventilation at preliminary testing. HR, maximum heart rate at preliminary testing. RER, maximum respiratory exchange ratio at preliminary resting.

* Significant main effect (*p* ≤ 0.05).

^C^ Significantly different from control group (*p* ≤ 0.05).

^E^ Significantly different from endurance‐trained athletes (*p* ≤ 0.05).

^S^ Significantly different from strength‐trained athletes (*p* ≤ 0.05).

The measurement of maximal performance revealed significantly (*p* < 0.05) higher absolute VO_2_max (4316 ± 554 (ET) vs. 3350 ± 515 (CG) and 3583 ± 767 mL · min^−1^ (ST)), higher relative VO_2_max (65.1 ± 4.8 (ET) vs. 41.2 ± 6.5 (CG) and 42.5 ± 4.7 mL · min^−1^ · kg^−1^ (ST)) and higher maximum ventilation (156 ± 26 (ET) vs. 118 ± 11 (CG) and 122 ± 23 L · min^−1^ (ST)) in ET compared to CG and ST. Maximum HR was only significantly (*p* < 0.05) different between CG and ST (186 ± 10 (ET), 196 ± 10 (CG) vs. 183 ± 15 bpm (ST)), while there were no differences in RER between the three groups (1.15 ± 0.05 (ET) vs. 1.14 ± 0.05 (CG) and 1.15 ± 0.06 VCO_2_ · VO_2_^−1^ (ST)). ET could therefore be characterized as having a higher aerobic capacity than the other groups (Table 1). There were no significant longitudinal changes of anthropometric and maximum performance data from preliminary testing. Mean group characteristics of participants are listed in Tables S1 and S2.

### 3.2 EE test

There were significant main effects (Table 2) between ET and CG in in all parameters (*p ≤* 0.05) excluding LA_105_ and RPE (*p* > 0.05). ET had a significantly higher running velocity at IANT compared with CG with corresponding significantly lower Lac concentrations (IANT) and significantly higher HRs (*p* ≤ 0.05). There was no significant difference in RPE (*p* > 0.05). There was a significant main effect on training phase in La_105_ (*p* ≤ 0.05) with a steady increase throughout the three consecutive training phases and three consecutive visits in ET and CG, respectively. ET had significantly higher calculated absolute and relative VO_2_ at IANT throughout the training year with significantly higher calculated MET at IANT. No interaction effect (group × training) in response to EE was detected (*p* > 0.05).

**TABLE 2:** Endurance exercise test.

	Visit	Control (CG)	Training phase	Endurance‐trained (ET)
		(*n* = 12)		(*n* = 10)
v_IANT_ (m · s^−1^)*	I	2.52 ± 0.34	PP	4.84 ± 0.35^C^
	II	2.62 ± 0.41	CP	4.86 ± 0.38^C^
	III	2.66 ± 0.45	RP	4.74 ± 0.40^C^
HR_IANT_ (bpm)*	I	160 ± 11	PP	172 ± 11^C^
	II	161 ± 12	CP	169 ± 11
	III	163 ± 15	RP	174 ± 12
La_95_ (mmol · L^−1^)*	I	2.8 ± 0.5	PP	2.0 ± 0.3^C^
	II	2.8 ± 0.7	CP	2.1 ± 0.4^C^
	III	3.0 ± 0.6	RP	2.2 ± 0.6^C^
La_100_ (mmol · L^−1^)*	I	3.1 ± 0.9	PP	2.5 ± 0.4
	II	3.4 ± 1.0	CP	2.7 ± 0.5
	III	3.7 ± 0.9	RP	2.8 ± 0.6^C^
La_105_ (mmol · L^−1^)	I	3.8 ± 1.4	PP	4.2 ± 0.7
	II	4.1 ± 1.4	CP	4.6 ± 1.0
	III	4.9 ± 1.1	RP	4.7 ± 1.2
HR_95_ (bpm)*	I	159 ± 11	PP	167 ± 11
	II	161 ± 13	CP	167 ± 8
	III	163 ± 12	RP	169 ± 12
HR_100_ (bpm)*	I	169 ± 11	PP	177 ± 11
	II	171 ± 13	CP	176 ± 9
	III	172 ± 13	RP	179 ± 13
HR_105_ (bpm)*	I	176 ± 10	PP	184 ± 11
	II	177 ± 12	CP	184 ± 10
	III	181 ± 12	RP	187 ± 12
RPE (6–20)	I	15 ± 2	PP	15 ± 2
	II	15 ± 2	CP	15 ± 2
	III	16 ± 2	RP	16 ± 2
VO_2 IANT_ (mL · min^−1^ · kg^−1^)* ^,^ ^a^	I	33.2 ± 5.8	PP	58.5 ± 5.6^C^
	II	34.0 ± 6.7	CP	58.6 ± 5.5^C^
	III	34.3 ± 6.7	RP	57.3 ± 5.6^C^
VO_2 IANT_ (% VO_2_max)* ^,^ ^a^	I	81.4 ± 6.3	PP	89.7 ± 4.3^C^
	II	82.8 ± 8.1	CP	90.0 ± 5.2^C^
	III	82.8 ± 8.0	RP	88.1 ± 6.0
MET_IANT_ (mL · min^−1^ · kg^−1^)* ^,^ ^a^	I	9.5 ± 1.6	PP	16.7 ± 1.6^C^
	II	9.7 ± 1.9	CP	16.7 ± 1.6^C^
	III	9.8 ± 1.9	RP	16.4 ± 1.6^C^

*Note*: All data are presented as mean ± SD.

Abbreviations: HR, heart rate at individual anaerobic threshold (IANT). La, blood lactate concentration at 95, 100 and 105% of individual anaerobic threshold (IANT). v, running velocity at individual anaerobic threshold (IANT). MET, metabolic equivalent at individual anaerobic threshold (IANT). # VO_2_ at IANT as well as MET_IANT_ calculated from preliminary test.

* significant main effect (*p* ≤ 0.05).

^a^ VO_2_ at IANT as well as MET_IANT_ calculated from preliminary test.

^C^ significantly different from control group (*p* ≤ 0.05). No training phases effects or effects of visits were detected.

### 3.3 RE test

There were significant main effects (Table 3) of absolute and relative strength performances as well as total work between ST and CG, with ST being substantially stronger and performed more work over all repetitions and sets than CG (*p* ≤ 0.05).

**TABLE 3:** Resistance exercise test.

	Visit	Control (CG)	Training phase	Strength (ST)	
		(*n* = 12)		(*n* = 12)	
MVIC (N)*	I	4001 ± 809	PP	6021 ± 1484^C^	
	II	3789 ± 768	CP	5871 ± 1791^C^	
	III	3749 ± 1096	RP	5656 ± 1326^C^	
MVIC_rel_ (N · kg^−1^)*	I	50 ± 11	PP	76 ± 14^C^	
	II	47 ± 12	CP	72 ± 16^C^	
	III	46 ± 12	RP	71 ± 14^C^	
MVC (N)*	I	4166 ± 803	PP	5629 ± 1285^C^	
	II	4253 ± 934	CP	5555 ± 1136^C^	
	III	3834 ± 930	RP	5311 ± 864^C^	
Total work (J)*	I	9186 ± 1887	PP	10,986 ± 2482	
	II	9124 ± 1898	CP	10,385 ± 2087	
	III	8649 ± 2073	RP	10,487 ± 1880^C^	

*Note*: All data are presented as mean ± SD.

Abbreviations: MVIC, absolute maximum voluntary isometric contraction. MVC, absolute maximum voluntary concentric contraction.

* Significant main effect (*p* ≤ 0.05).

^C^ significantly different from control group (*p* ≤ 0.05). No training phases effects or effects of visits were detected.

### 3.4 Exercise‐induced changes of the metabolic profile

Multivariate analysis with PLS‐DA revealed significant statistical models of exercise‐induced effects between the groups and training phases. Corresponding univariate analysis of exercise‐induced effects in the corresponding training phases and visits are reported in Tables S3 and S4.

**FIGURE 2:**
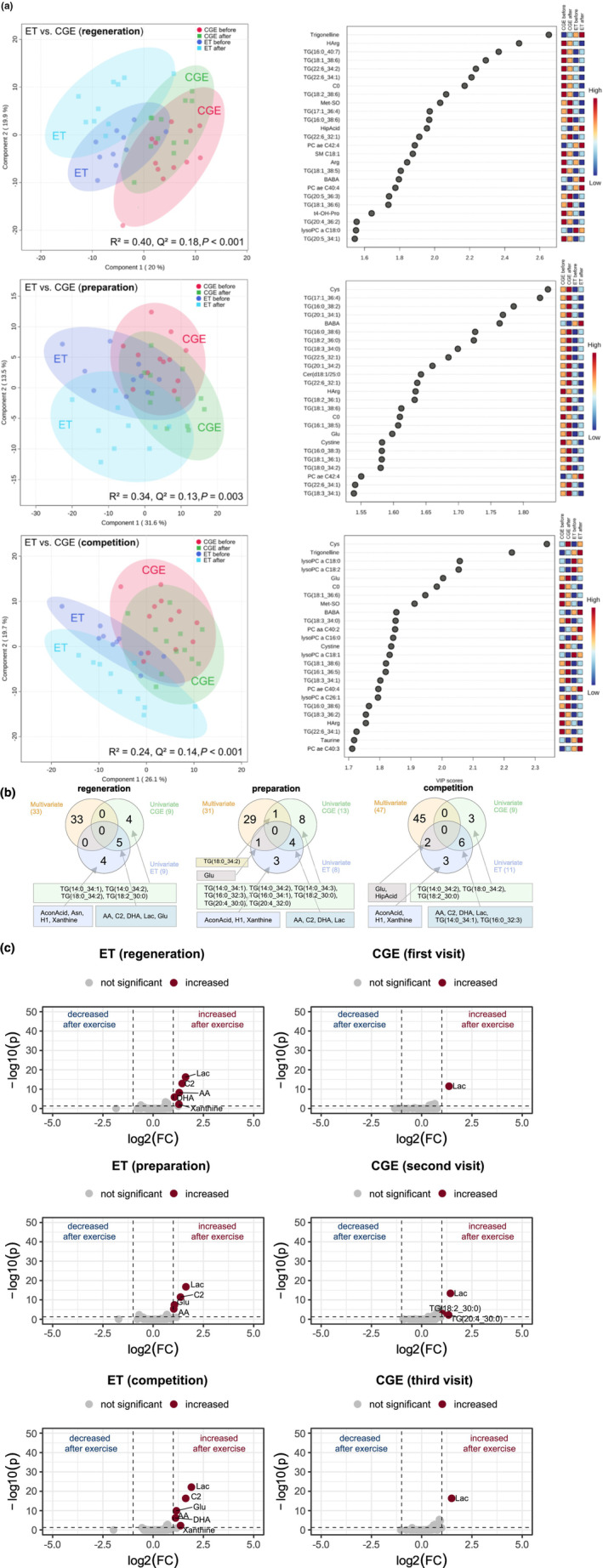
(a) PLS‐DA analysis with corresponding Top 25 VIP scores of exercise‐induced metabolites of endurance‐trained (ET) athletes compared with endurance trial of control group (CGE) in the three training phases; top, regeneration phase (1. visit in CGE); middle, preparation phase (2. visit in CGE); bottom, competition phase (3. visit in CGE). (b) Venn diagrams in the different training phases or respective visits for simultaneous comparison of uni‐ (ET before vs. after, CGE before vs. after) and multivariate analysis. (c) volcano plots depicting metabolomic diversity in each training phase in ET and CGE. Each point represents a metabolite. Darkblue and darkred indicate significantly (above dotted horizontal line) decreased (log2(FC) ≤ −1.0, left side of dotted vertical lines) or increased (log2(FC) ≥ 1.0, right side of dotted vertical lines) metabolites after exercise, gray indicates nondifferential metabolites.

PLS‐DA of exercise‐induced changes over the three training phases in ET as well as CGE (regeneration: *R* ^2^ = 0.40, *Q* ^2^ = 0.18, *p* < 0.001; preparation: *R* ^2^ = 0.34, *Q* ^2^ = 0.13, *p* < 0.001; competition: *R* ^2^ = 0.24, *Q* ^2^ = 0.14, *p* < 0.001) showed a robust discrimination with low to moderate predictive ability (Figure 2a).

In the RP or the respective visit in CGE, venn‐diagrams revealed no simultaneously in multi‐ and univariate analysis changed metabolites in ET and CGE. Univariate analysis revealed five metabolites (AA, C2, DHA, Glu, Lac) as significantly increased after exercise in ET and CGE. Some metabolites were changed solely in ET (AconAcid, Asn, H1, xanthine) or in CGE [TG(14:0_34:1), TG(14:0_34:2), TG(18:0_34:2), TG(18:2_30:0)] (Figure 2b). In addition, there was no overlap between multivariate and univariate analysis in CGE.

The preparation phase (respective visit in CGE) was characterized by one metabolite being present in multivariate and univariate analysis in ET (Glu) and one metabolite being present in multivariate and univariate analysis in CGE [TG(18:0_34:2)]. In univariate analysis, four metabolites were detected with significantly increased concentration after exercise in both groups (AA, C2, DHA, Lac). Three metabolites were only significantly increased in ET (AconAcid, Asn, H1), eight triglycerides [TG(14:0_34:1), TG(14:0_34:2), TG(14:0_34:3), TG(16:0_32:3), TG(16:0_34:1), TG(18:2_30:0), TG(20:4_30:0), TG(20:4_32:0)] only in CGE.

The CP (respective visit in CGE) was characterized by two metabolites being present in multivariate and univariate analysis in ET (Glu, HipAcid) but not in CGE. In addition, there was no overlap between multivariate and univariate analysis in CGE. Univariate analysis revealed three significantly increased metabolites in CGE [TG(14:0_34:2), TG(18:0_34:2), TG(18:2_30:0)], six metabolites in both groups, CGE and ET (C2, DHA, Lac, TG(14:0_34:1), TG(16:0_32:3), AA) and three metabolites only in ET (AconAcid, Asn, H1).

Only a few metabolites could be declared as robust with significant and high fold‐changes after exercise in either ET or CGE or in both groups (Figure 2c). In ET there were three metabolites, which were increased in each of the three training phases: lactic acid (Lac, FC = 1.6–1.9, *p* < 0.001), acetylcarnitine (C2, FC = 1.4–1.6, *p* < 0.001) and arachidonic acid (AA, FC = 1.0–1.3, *p* < 0.001). Docosahexaenoic acid (DHA), (FC = 1.1, *p* < 0.001) and xanthine (FC = 1.3–1.4, *p* < 0.01) were only increased after exercise in the regeneration and CP, but not in preparation phase. Glutamate (Glu), (FC = 1.1–1.2, *p* < 0.001) was only increased in the preparation and CP, but not in resting period.

In CGE, only lactic acid (Lac, FC = 1.4–1.5, *p* < 0.001) was increased after each EE test. During the second visit only, an increase in some triglycerides TG(18:2_30:0) [FC = 1.0, *p* < 0.01] and TG(20:4_30:0) [FC = 1.4, *p* < 0.01] was observed after the EE test. Overall, no metabolites were found to be decreased after exercise.

**FIGURE 3:**
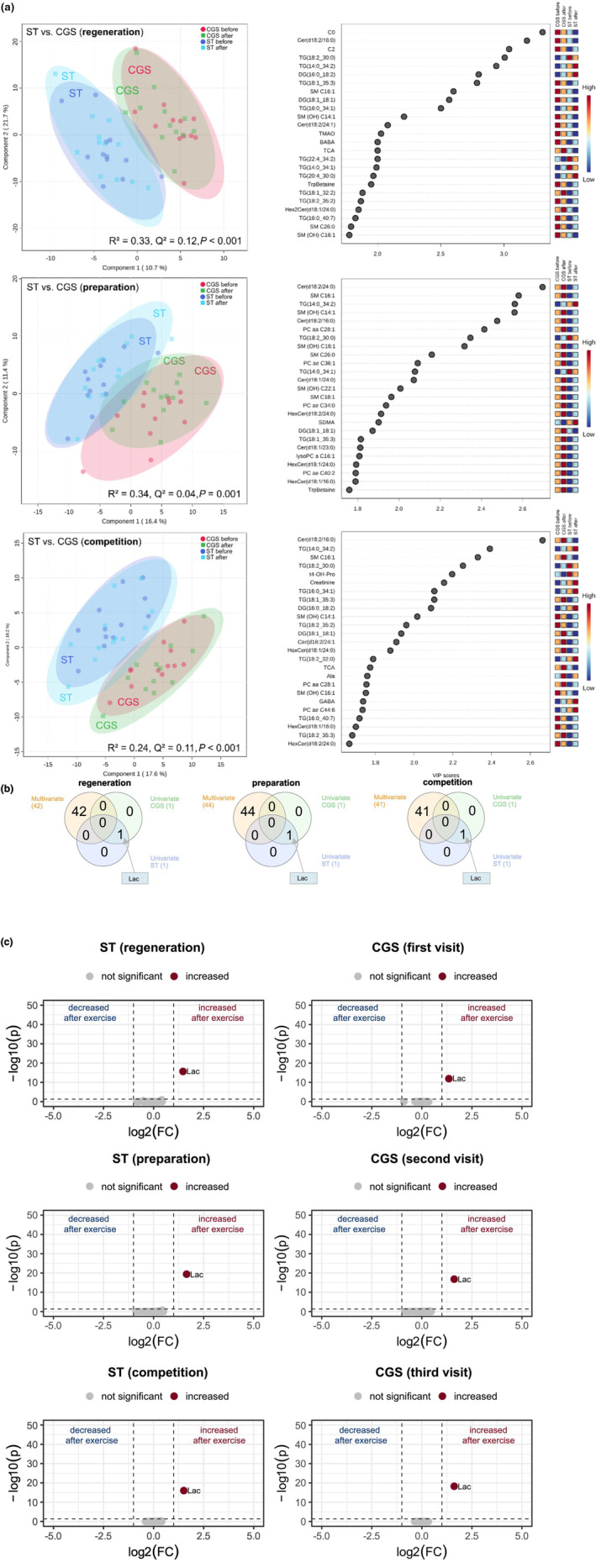
(a) PLS‐DA analysis with corresponding Top 25 VIP scores of exercise‐induced metabolites of strength‐trained (ST) athletes compared with strength trial of control group (CGS) in the three training phases or respective visits; top, regeneration phase (1. visit in CGS); middle, preparation phase (2. visit in CGS); bottom, competition phase 3. (visit in CGS). (b) Venn diagrams in the different training phases or respective visits for simultaneous comparison of uni‐ (ST before vs. after, CGS before vs. after) and multivariate analysis. (c) volcano plots depicting metabolomic diversity in each training phase in ST and CGS. Each point represents a metabolite. Darkblue and darkred indicate significantly (above dotted horizontal line) decreased (log2(FC) ≤ −1.0, left side of dotted vertical lines) or increased (log2(FC) ≥ 1.0, right side of dotted vertical lines) metabolites after exercise, gray indicates nondifferential metabolites.

PLS‐DA of exercise‐induced changes over the three training phases in ST as well as CGS (regeneration: *R* ^2^ = 0.33, *Q* ^2^ = 0.12, *p* < 0.001; preparation: *R* ^2^ = 0.34, *Q* ^2^ = 0.04, *p* < 0.001; competition: *R* ^2^ = 0.24, Q^2^ = 0.11, *p* < 0.001) showed a robust discrimination with low predictive ability (Figure 3). Venn‐diagrams revealed, that no metabolites were simultaneously changed in multi‐ and univariate analysis in CGS and ST in all training phases. There were only minor changes in univariate analysis with only one significantly increased metabolite. Lactic acid (Lac) was increased after exercise in each phase in ST (FC = 1.5–1.7, *p* < 0.001) as well as in CGS after the strength test (FC = 1.4–1.6, *p* < 0.001) (Figure 3).

**FIGURE 4:**
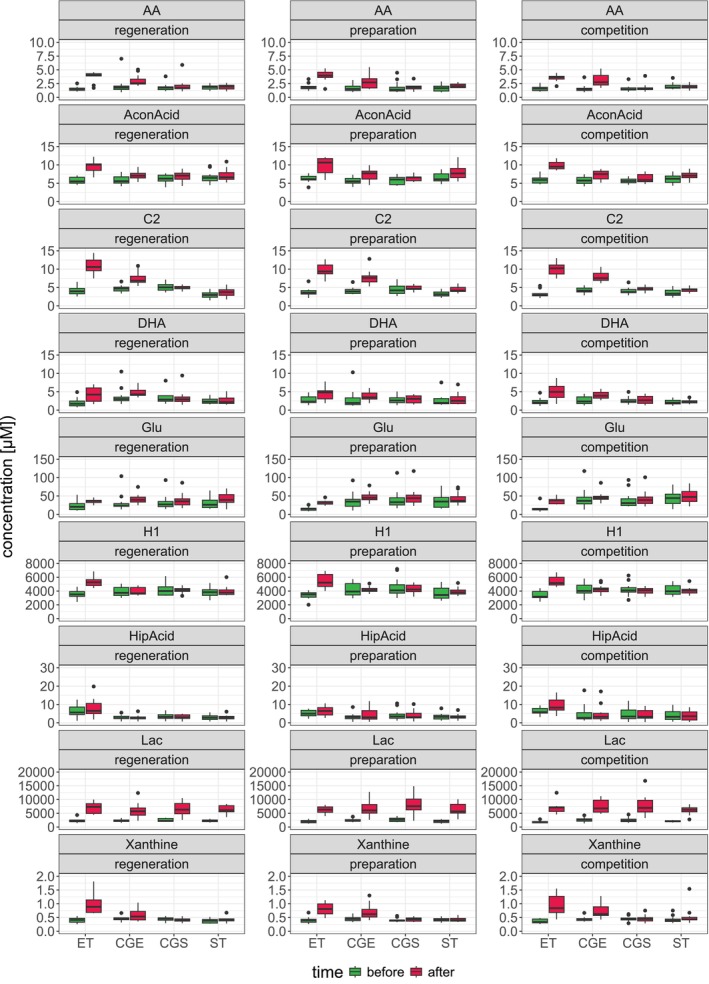
Box plots with the most changed metabolite concentrations before (green) and after (red) corresponding exercise in each group (ET, CGE, CGS, ST) and training phase (regeneration, preparation, competition in ET and ST) or respective visits (first, second, third visit in CGE and CGS). Most changes occurred after endurance exercise test in ET followed by CGE. Detailed data and metabolite abbreviations are summarized in Tables S3 and S4. The boxes range from the 25% and the 75% percentiles; the 5% and 95% percentiles are indicated as error bars; single data points are indicated by circles. Medians are indicated by horizontal lines within each box.

Overall, there were the highest number of metabolic changes in ET, followed by CGE, ST and CGS. In Figure 4 all significantly changed metabolites are shown. Most of the significant changes were found in ET after the endurance exercise test.

## 4 DISCUSSION

The present study was designed to investigate characteristic responses of the plasma metabolome to standardized specific exercise stimuli in endurance‐ or strength‐trained athletes in comparison to the metabolic responses to these specific exercise stimuli in sedentary subjects. To our best knowledge, for the first time, plasma metabolome was studied in strength‐trained or endurance‐trained athletes during 1 year in periods of preparing for competition, competing, or regenerating and compared with the findings in sedentary subjects in response to characteristic, standardized exercise stimuli. In summary, we observed significant differences in the metabolomes at rest and after the specific exercise stimuli (i) between endurance‐trained athletes and sedentary subjects, (ii) between strength‐trained athletes and sedentary subjects (iii) between endurance‐ and strength‐trained athletes. The exercise‐induced effects on the metabolome were more pronounced after EE than after RE. Only Lac was significantly increased after both types of exercise in the endurance‐ or strength trained athletes and in the sedentary controls, and lactate was the only significantly increased metabolite after RE. Besides lactate, three metabolites pointing to altered lipid metabolism [acylcarnitine (C2), arachidonic acid (AA), DHA] were significantly increased after EE in endurance‐trained athletes and in sedentary controls. The significant exercise‐induced increase of xanthine in endurance‐trained athletes only might indicate a depletion of high‐energy phosphate compounds. The significant increase in Glu in this group of athletes only suggests an involvement of the protein metabolism. Surprisingly, we did not observe any significant changes with regard to the different training periods.

In contrast to most other studies on the exercise‐induced effect on the metabolome we chose exercise stimuli of short duration. As endurance‐exercise stimulus a treadmill test was performed which is regularly applied in endurance‐trained athletes to evaluate the running velocity at the maximal lactate steady state. Continuous and interval training with running velocities approximating the maximal lactate state have been shown to be an efficient stimulus for the improvement of the aerobic performance capacity (Philp et al., 2008). We observed higher HR with a lower lactate concentration at IANT in endurance athletes compared to the sedentary subjects (Table 2). Additionally, endurance athletes performed the threshold run with a substantially higher metabolic equivalent but with identical perceived exertion compared with control subjects. Thus, endurance athletes had a higher metabolic activation and energy expenditure than the control subjects.

Strength‐trained athletes had higher absolute and relative dynamic strength and were stronger than controls in response to strength exercise (Table 3). Despite the fact, that the individuals performed all repetitions with maximum intention and same dynamic velocities total work was higher in strength athletes and therefore metabolic activation and energy turnover was higher in strength athletes compared to the sedentary subjects.

### 4.1 Exercise‐induced changes of the metabolic profile

Multivariate analysis with PLS‐DA revealed significant statistical models of exercise‐induced effects between the groups and training phases, with the most robust discrimination and low to medium predictive ability between ET and CGE, while there was a less robust discrimination and the lowest predictive ability between ST and CGS as well as between both groups of athletes. Overall, only very few overlaps of metabolites in the multi‐ and univariate analyses were observed: Glu and hippuric acid (HipAcid) in the comparison of ET vs. CGE and acetylcarnitine (C2). Furthermore, C2, Glu and Xanthine were the metabolites with the highest fold‐changes in univariate analysis and could therefore be stated as the most robust metabolites.

Lactate (Lac) was significantly increased after exercise in all groups and time points but was not found to be a discriminating metabolite between the groups of athletes and controls. Lactate (Lac) is an already established performance diagnostic marker of energy metabolism and at the same time the most frequently studied exercise‐induced metabolite (Beneke et al., 2011; Brooks, 2018). During high‐intensity exercise, available blood glucose and muscle glycogen are the primary sources of energy production (Romijn et al., 1993; van Loon et al., 2001). Activated glycolysis leads to an increase in pyruvate or lactate (Schranner et al., 2020). In the present study, the observed increases in lactate were slightly lower (2.6–3.2‐fold after EE and 2.9‐fold after RE, respectively) than the values reported in a recent review (Schranner et al., 2020) (3‐ to 8‐fold increases), probably explained by the different exercise protocols. In two studies investigating in the effects of endurance and RE (Morville et al., 2020; Pellegrino et al., 2022) although with longer duration of both exercise bouts, significant and similar increases in lactate were found after both types of exercise. In the present study, lactate was repeatedly measured in capillary blood obtained from the earlobe during EE. The increase in capillary blood lactate was similar (2.9–3.3‐fold) to the values determined in the metabolomics analysis (2.6–3.2‐fold).

The non‐significant exercise‐induced increases in hexoses in ET only (1.5–1.6‐fold) point to the activated carbohydrate metabolism in contrast to sedentary subjects and strength athletes, probably a result of the perennial endurance training. In, to our best knowledge, the only other study on exercise‐induced effects on the blood metabolome with similar exercise duration, a 1.3‐fold increase in glucose, the most important hexose in the carbohydrate metabolism, only in endurance athletes in comparison with sprinters and natural body builders was observed (Schranner et al., 2021). Blood glucose was also significantly increased after a marathon race (Stander et al., 2018) and after a graded exercise test on a cycle ergometer lasting 1 h as well as (to a somewhat lower extent) after an aerobic training session over 5:20 h (Nemkov et al., 2023). Increased hexose concentrations were found in fitter subjects after a marathon race (Schader et al., 2020). The increased concentration in hexoses may be explained by hepatic glucose production via glycogenolysis and gluconeogenesis (Brooks, 2020; Kjær, 1998). An increased hepatic glycogenolysis is primarily observed during short term high‐intensity exercise, while gluconeogenesis accounts for 25%–50% of total glucose production after low to moderate intensity and long duration exercise (Kjær, 1998).

The non‐significant increases in the carboxylic acids, aconitic acid (AconAcid) and in hippuric acid (HipAcid), in ET only (Figure 2 and Figure 4) probably indicate exercise‐induced effects on the tricarboxylic acid cycle in the endurance athletes (Nemkov et al., 2023; San‐Millán et al., 2020) which is central in carbohydrate and lipid metabolism.

EE stimulates lipolysis in fat tissue as well as fat oxidation within the mitochondria (Lundsgaard et al., 2018) leading to increased acylcarnitine concentrations in blood (Schranner et al., 2020). We observed significantly increased concentrations of acetylcarnitine (C2) after EE in endurance‐trained athletes and in sedentary subjects in univariate analysis, but no changes in all other acylcarnitines (Figures 2 and 4). This finding is in accordance with the increase in short‐chain acylcarnitines reported after a cycle ergometry with similar duration as the EE in the present study (Schranner et al., 2021). There is some evidence for elevated concentrations in quite a number of medium and long‐chain acylcarnitines after long‐lasting EE, especially in endurance athletes (Howe et al., 2018; Lehmann et al., 2010; Nemkov et al., 2023; San‐Millán et al., 2020; Schader et al., 2020) indicating increasing lipid oxidation with exercise duration. The non‐significant increase in several triglycerides after EE in sedentary subjects suggests stimulated lipolysis in fatty tissue. It might be assumed that endurance athletes rely more on fatty acids stored within the skeletal muscle not leading to increased triglyceride concentrations in the blood.

The polyunsaturated fatty acids (PUFAs), arachidonic acid (AA; 20:4ϖ‐6) and DHA (DHA; 22:6ϖ‐3) were significantly increased after EE in endurance‐trained athletes (AA 1.0‐fold to 1.2‐fold in all training phases, DHA 1.6‐fold to 2.2‐fold) and in sedentary subjects (AA 1.4‐fold to 1.9‐fold in all training phases, DHA 1.3‐fold to 1.5‐fold) (Figure 2 and Figure 4). Significant increases in these PUFAs have also been observed—among significant increases of other fatty acids—after endurance exercise of longer duration than in the present study (Nemkov et al., 2023; San‐Millán et al., 2020) with no difference between high‐performance cyclists and cyclists of a somewhat lower performance level in the response to the exercise stimulus (San‐Millán et al., 2020). Some studies only reported a significant increase in DHA after long lasting exercise among significant increases of other fatty acids, but no increase in AA (Howe et al., 2018; Schranner et al., 2020). The implication of significant increase of AA and DHA even after endurance exercise of rather short duration as in the present study is not known. Both PUFAs might play a role in exercise‐induced muscle‐damage and inflammation (Davinelli et al., 2019; Heileson JL, Harris DR, Tomek S, Ritz PP, Rockwell MS, Barringer ND, Forsse JS, Funderburk LK, 2023; Ochi & Tsuchiya, 2018). However, repeated exercise‐induced increases in these PUFAs might have effects beyond the already mentioned effects on skeletal muscle. AA pathways play a key role in cardiovascular biology, several inflammatory processes and carcinogenesis (Wang et al., 2021). DHA is a critical component of lipid structures with effects on signaling, especially neuronal signaling, inflammatory processes, insulin sensitivity, glucose and fatty acid metabolism (Calder, 2008).

The only metabolite with a significantly different EE‐induced response between endurance‐trained athletes and sedentary subjects was Glu with a significant increase in the preparation (2.1‐fold) and competition period (2.2‐fold). The results of metabolomic studies on the exercise‐induced response of Glu are equivocal (Schranner et al., 2020) with increased (Schader et al., 2020), unchanged (San‐Millán et al., 2020) or decreased Glu concentrations (Howe et al., 2018; Nemkov et al., 2023) after long‐lasting endurance exercise. Dynamic exercise changes whole‐body muscle protein turnover and thus amino acid concentration (Gibala, 2007; Rennie & Tipton, 2000). There is evidence that high‐intensity exercise changes amino acid concentration of Glu and alanine (Ala) with concomitant increases in ammonia in proportion to exercise intensity (Al‐Khelaifi et al., 2019; Rennie, 2011). The Glu‐glutamine cycle is of particular importance as a large part of the nitrogen produced in the muscles is removed via glutamine (Gln) (Newsholme et al., 2003). Glu appears to have an important role in the transfer of amino groups as well as in the citrate cycle (Mourtzakis & Graham, 2002). In the basal metabolome, we observed significantly reduced Glu levels in endurance‐trained athletes in comparison with strength‐trained athletes and with sedentary subjects (Parstorfer et al., 2023). Glu is primarily found intracellularly with limited ability to leave the cell (Newsholme et al., 2003). Apparently, short‐term endurance exercise triggers Glu release in endurance‐trained athletes probably of importance for the removal of ammonia produced during longer lasting endurance exercise. We found a significant 2.3‐fold upregulation of the ATP breakdown product xanthine in the group of endurance athletes in response endurance exercise in the regeneration and CP, while there were no significant changes in the concentration of xanthine in the CG in all phases (Figure 2). Increased levels of ATP breakdown products (Xanthine, Hypoxanthine, Xanthosine) were reported after both, endurance and RE (Howe et al., 2018; Morville et al., 2020; Pellegrino et al., 2022; San‐Millán et al., 2020). Increased ATP turnover, e.g., in response to short, high‐intensity or prolonged exercise, is linked to a rise in adenine nucleotide metabolites (inosine‐5′‐monophosphate, inosine). These metabolites accumulate in skeletal muscle or efflux into the blood where they are further metabolized to hypoxanthine and xanthine via the purine nucleotide cycle (Lowenstein, 1978; Zieliński et al., 2019). Surprisingly, we found no differences in the ATP breakdown product xanthine in the group of strength‐trained athletes or in the CG after the strength exercise. The nearly unchanged plasma metabolome after RE in strength‐trained athletes and in sedentary subjects is striking (Figures 3 and 4). Maximal strength tests which are usually applied in performance diagnostic were chosen as RE in order to mimic the short bursts of intense muscle contraction which are typical in weightlifting. Certainly, the metabolic cost of the RE was much lower compared with the endurance exercise. However, the significant increase in lactate indicates that changes in the metabolome occurred during the RE of short duration. There must have been further changes, e.g., in the purine metabolism and probably in protein breakdown. The reason for the lack of change in these metabolites in plasma remains to be elucidated. It might be speculated that, due to the short duration of the RE, the accumulation of the breakdown products was not sufficient for a significant efflux from the skeletal muscle to the blood and that the breakdown products might have been subject to resynthesis within the skeletal muscle. In two studies which reported significant responses of the plasma or serum metabolome to RE, the subjects performed a bout of strength training which lasted about 1 h and was of similar duration as the endurance exercise in these studies (Morville et al., 2020; Pellegrino et al., 2022). While one study found fairly robust responses of the serum metabolome to RE and to endurance exercise (Pellegrino et al., 2022), another study reported some differences in cluster analyses between both types of exercise immediately after and during the first hours of recovery from exercise (Morville et al., 2020). Only modest correlations between muscle and plasma metabolite levels observed in a study on strength training effects in healthy older subjects and frail older subjects indicate that not all changes in the muscle metabolome are reflected in analyses of the plasma metabolome. The discrepancy between muscle and plasma metabolome might, at least partly, explain the unexpected findings after RE of the present study needs further investigation (Fazelzadeh et al., 2016).

### 4.2 Exercise‐induced responses of the metabolome over 1 year of training

There were only minor changes in the exercise‐induced response of the metabolome in the different training periods. These changes do not allow conclusions with regard to an altered exercise metabolism reflecting differences in the varying number, frequency, duration and intensity of training sessions.

### 4.3 Limitations

The generalizability of these results is subject to certain limitations. A notable limitation of our study is the disparity in the metabolic stressors between the endurance and RE protocols. While EE involved sustained aerobic effort, RE consisted of brief maximal contractions, which may have led to a localized metabolic response not fully captured in plasma. This difference in exercise modality may have influenced the extent of metabolomic changes observed. Additionally, the lack of crossover between the athlete groups (i.e., ET not performing RE and vice versa) limits the ability to make direct comparisons between the systemic metabolic responses between the athletes. For instance, skeletal muscle is a huge source of metabolically active tissue in human body (Fazelzadeh et al., 2016). Plasma metabolite concentrations only partially reflect muscle metabolism as several metabolites are produced by other metabolic compartments and could not be used as a direct read‐out of muscle metabolism (Fazelzadeh et al., 2016). Accordingly, a recent study found no correlation between acylcarnitine concentrations in plasma and tissues in mice, which was due to differences in turnover between blood plasma and muscle compartments as well as other compartments than muscle (Schooneman et al., 2014). The standardization of training and testing sessions in the field of high‐performance sports with its low subject numbers is another challenge. The subject number of the present study was similar to the subject numbers of other investigations on acute effects of endurance or strength exercise on the metabolic profile of athletes. These studies did not comment on the resulted statistical power. However, number of subjects might have been too low to achieve sufficient statistical power for group comparisons specified in our study by the model performance indicators of multivariate analysis. Supervised statistical analysis tend to overfit the data. We did not include data that was overfitted and strictly excluded them from further analysis, thus our metabolite set needs to be validated in an independent sample or a sample divided into a training and test set. Whether our results are transferable to female athletes remains to be elucidated. There could also be an impact of time‐varying factors as we studied the subjects over 1 year of training. For example, due to a rescheduling of the initially planned main competition in the group of weightlifters of several weeks, it was not possible to study both groups of athletes with the same time span between the trials. Another weakness of this study that may have affected the metabolome was the non‐fasted state of the subjects and their chronic dietary and supplementation habits. To compensate for the non‐fasted state, the subjects could choose one of three pre‐meals with the same relative energy and macronutrients distribution as mentioned above (Parstorfer et al., 2023).

### 4.4 Perspectives and significance

In the realm of sports medicine, our study sheds light on athlete's metabolism in response to exercise‐specific stimuli. While a bout of endurance exercise induced similar significant increases in some metabolites of the lipid metabolism and the oxidative defense system in endurance trained athletes and sedentary subjects, also signs of distinct differences between these subject groups in the exercise‐induced changes of the plasma metabolome were observed. However, no clear variations in the altered specific metabolites were observed throughout the training year. Therefore, these metabolites might provide some clues on the training status but cannot easily be applied for monitoring athletic performance. The RE bout of the present study did, besides a significant increase in lactate, not induce changes of the plasma metabolome. Probably, the duration of the exercise bout was too short. However, RE aiming at the development of maximal explosive strength, is of short duration and effectively stimulates energy metabolism. Probably, characteristic changes of metabolome might only be found within the skeletal muscle and not be reflected by changes in plasma metabolites.

## AUTHOR CONTRIBUTIONS

Conceptualization, B.F.‐B. and M.P.; methodology, B.F.‐B., M.P., G.P. and K.B.; software, G.P. and M.P.; formal analysis, M.P., G.P. and D.K.; investigation, G.P. and M.P.; resources, M.P.; data curation, M.P.; writing—original draft preparation, M.P. and B.F.‐B.; writing—review and editing, M.P. and B.F.‐B.; visualization, M.P.; supervision, B.F.‐B.; project administration, B.F.‐B. and M.P.; funding acquisition, B.F.‐B. and M.P. All authors have read and agreed to the published version of the manuscript.

The study was conducted in accordance with the Declaration of Helsinki and approved by the Ethics Committee of the University of Heidelberg for studies involving humans (protocol code S513/2018 and approved on 3 September 2018). Written informed consent has been obtained from the subject(s) to publish this paper.

## FUNDING INFORMATION

This study was funded by Grant No. ZMVI4‐070101/18–20 from the German Federal Institute of Sports Science, Bonn, Germany. We thank the Metabolomics Core Technology Platform of the CellNetworks Core Technology Platform, University Heidelberg University, Heidelberg, Germany for support with metabolite quantification. The authors gratefully acknowledge the data storage service SDS@hd supported by the Ministry of Science, Research and the Arts Baden‐Württemberg (MWK) and the German Research Foundation (DFG) through grant INST 35/1314–1 FUGG and INST 35/1503–1 FUGG.

## CONFLICT OF INTEREST STATEMENT

No conflicts of interest, financial or otherwise, are declared by the authors.

## Supporting information


Table S1.

Table S2.

Table S3.

Table S4.

## Data Availability

Data will be made available upon reasonable request by the corresponding author. Data is not publicly available due to privacy or ethical restrictions. The custom‐made data analysis code for R is available from the corresponding author upon reasonable request.
